# London Empty

**Published:** 1889-10-05

**Authors:** 


### The Hospital

A Weekly Institutional Journal of

**Science, Medicine, Nursing, and Philanthropy.**

*For Hospitals, Asylums, Medical Practitioners, Students, Nurses, Families, Parishes, Congregations, and the Charitable Public.*

Vol. VII. No. 158.] [October 5, 1889.

### "LONDON EMPTY."

*Punch*, the most wholesome of satirists, has given his readers, week by week for the last few weeks, a series of pictures of "London Empty." Beginning with The Angel, in the North, with its pandemonium of houses, "busses," cabs, tramcars, men, women, policemen, horses, dogs, costermongers, peripatetic and stationary "tray" merchants, shouts, curses, laughter, songs, and all the indescribable *melée* of rushing and eager, yet ordered and purposed life, he went on to present a scene of the same kind at The Elephant and Castle, in the South, a third at Whitechapel, in the East, and a fourth in a certain "unknown" and "unclassical" yet equally crowded, eager, and, if possible, more "slummy" region in the West. The obvious moral is that, though for certain persons who choose to regard themselves as "the world" London is "empty," yet from the point of view of the statesman, the practical municipal ruler, the philosopher, the historian, and the moralist, the huge creature which constitutes the true London has but shed a few of its more or less ornamental hairs. Itself remains where it always is—grinding in its ceaseless mill, laughing in its prison, wallowing in its mire.

The man who wishes to see and know London as it is must have seen and known, at least in imagination, the other great cities of the present day, such as Paris, Vienna, Manchester, Glasgow, and the like, and also the vast agglomerations of human beings in central places which constituted the crowded cities of past ages. What did those human creatures do from day to day; what did they think? Did they go forward in material prosperity; did they rise in morals; were they lighthearted and hopeful? Or did they strive in vain for the means of healthy life; did they relieve the tedium of toil by senseless and brutal amusements; despairing of success, did they sink down into a feeble and hopeless imbecility of character which, whilst it made government comparatively easy, made moral and material progress impossible? No man can even begin to comprehend London who does not possess a deep personal knowledge of the different classes who constitute it, and of their daily way of life. What is their work? What are their homes? How do they amuse themselves? Above all, what do they think in their hearts, of themselves, of their employers, of their "betters," of their rulers, of the present, of the future, of human nature and its destiny—here and hereafter? When the fight for daily bread is over, religion and politics engross the ordinary activities of the average British man. What is the religion, what are the politics, municipal and imperial, of the normal, typical, home-bred Londoner?

Any man of average intelligence, whose business has required him to pass through the streets of London for a few years, may easily discover that the hive of the metropolis contains a certain number of bees who are busy all the year round. They know no cessation of toil from the beginning to the end of the year, nor hardly from birth to death. Those human bees acquire a look, not of hopelessness, nor of despair, but simply of routine, fixed and changeless routine. The whole world of their comprehension is included in their little daily round. For them love and hate, poetry and madness, heaven and hell, have no existence. Like the mill-horse, they go round, stop for meals, cease work at bedtime, sleep until morning, and then resume, to repeat the same thing over again next day, and until the last day of their working lives. What proportion of the middle and lower classes do men and women of this stamp bear to the whole? Are they so many as half, or are they more? It is impossible to say. What sort of material are these to work upon for the philosopher of development, the statesman ambitious of noble citizens, the preacher who believes in living souls and tremendous destines? Rather hopeless, it is to be feared! Yet if half of the homebred Londoners are of this class, we have to face the fact of two millions of persons in London alone who, from the patriot's and the preacher's points of view, are so much dead-weight; or even worse than dead-weight, so much dead and decaying moral and spiritual matter.

But there is a worse aspect of "Empty London" even than that. A certain number of men and women, either naturally of the animal type, or forced down by the pressure of circumstances and despairing of ever finding their way back to wholesome and hopeful ways of living, have turned themselves finally into beasts of prey, and live, from choice, by robbery. To the majority of persons the "criminal class" is but a name—a name that suggests practically nothing. But what is a cancer in a vital organ of the human body? It is an abnormal growth that invades slowly but surely the sound and healthy parts surrounding it. In no long time the victim becomes aware that he is in the grasp of an enemy which will not relax its hold until life itself is destroyed. The criminal classes are the despairing classes. They despair of themselves; they despair of their honester fellows. Nothing is more contagious than despair. It is well known that every year hundreds succumb to the counsels of the tempter from mere contact. If we had no debauched and criminal class with us, it would be a comparatively easy task to preserve the honesty of those who are merely weak. But because they are in daily and hourly contact with desperate men, they are drawn over the precipice to their own ruin, and the injury of all other classes. London is never empty of its criminals. In summer as in winter, in autumn as in spring, they are always here; always leavening with their deadly poison the weak and the ignorant around them.

Now these two millions of routine bees, and these tens of thousands of the criminal classes, can understand object-lessons, and most of them can understand lessons of no other kind. If they read, they read for amusement, not for instruction. If they listen—as they probably do sometimes—to the lecturer and the preacher, they have neither the mental capacity to comprehend the scope and force of his arguments, nor the energy to apply them in daily life. But object-lessons the average Londoner very readily perceives and understands. It is amusing as well as surprising how soon the newest fashions in the west find their way north, south, and east, and are imitated in pathetic caricature by every "Arriet" of the sweating dens and every "Arry" of the slums. Whether it be for good or for evil, the rich and the prosperous set all the fashions. Not only do they set the fashions in dress, but in eating, in drinking, in dancing, in mourning, in praying, in swearing. They have leisure in which to acquire tastes and the means of indulging them when acquired. Other people have not, and so they take their tastes and their habits at second-hand.

But it is no light thing to be a visible rule of life and an embodied conscience for five millions of fellow-creatures. The people who are out of town have left behind them those who will not only imitate their tricks of dress and speech, but who will adopt their tone of mind. If a man is placed in the position of a finger-post to large numbers of his fellows, he owes it to them and to himself, if not to a higher, to point them in the right direction. That direction need not necessarily lead towards Puritanism, but it should lead at least to a wholesome, honest, courageous, and brotherly manhood. The rich who are feeble-minded and vain, or who are sordid and brutal, often bring greater injury upon their country than the debauched and the criminal. Everyone looks upon the great and the powerful as lights to guide; but the debauched and the criminal are regarded as beacons to warn. A false harbour light will wreck the ship, whilst a beacon will warn her from the rocks.

Though, then, the rich have fled from London, they have not fled from humanity and its imperative claims. They have temporary leisure and ease in order that they may have permanent thought and purpose. That many of them are despicably frivolous and worthless is, unhappily, too true. But many also are English in the best sense; narrow perhaps, prejudiced certainly, and wanting in universality of comprehension, if not of charity, yet imbued and commanded by as noble a sense of duty as ever was Roman warrior or martyr in the Christian fight. The duty that is laid upon them is nothing less than that of leading the noblest race the world has ever yet seen, and showing that noble race, by an object lesson, how it ought to live. Those of them who are made of right worthy stuff will not forget the five millions of their fellow citizens who must toil in the autumn as well as in the spring. The rich need London for the exercise and development of their best powers as much as London needs them for the help and guidance they can give. It is, perhaps, true indeed that London could better spare them than they spare London.

